# Enzymes from Fishery and Aquaculture Waste: Research Trends in the Era of Artificial Intelligence and Circular Bio-Economy

**DOI:** 10.3390/md22090411

**Published:** 2024-09-10

**Authors:** Zied Khiari

**Affiliations:** National Research Council of Canada, Aquatic and Crop Resource Development Research Centre, 1411 Oxford Street, Halifax, NS B3H 3Z1, Canada; zied.khiari@nrc-cnrc.gc.ca

**Keywords:** fishery and aquaculture waste, fish, shellfish and marine animals, enzymes, enzyme-producing microorganisms, substrate, zero-waste, valorization, artificial intelligence

## Abstract

In the era of the blue bio-economy, which promotes the sustainable utilization and exploitation of marine resources for economic growth and development, the fisheries and aquaculture industries still face huge sustainability issues. One of the major challenges of these industries is associated with the generation and management of wastes, which pose a serious threat to human health and the environment if not properly treated. In the best-case scenario, fishery and aquaculture waste is processed into low-value commodities such as fishmeal and fish oil. However, this renewable organic biomass contains a number of highly valuable bioproducts, including enzymes, bioactive peptides, as well as functional proteins and polysaccharides. Marine-derived enzymes are known to have unique physical, chemical and catalytic characteristics and are reported to be superior to those from plant and animal origins. Moreover, it has been established that enzymes from marine species possess cold-adapted properties, which makes them interesting from technological, economic and sustainability points of view. Therefore, this review centers around enzymes from fishery and aquaculture waste, with a special focus on proteases, lipases, carbohydrases, chitinases and transglutaminases. Additionally, the use of fishery and aquaculture waste as a substrate for the production of industrially relevant microbial enzymes is discussed. The application of emerging technologies (i.e., artificial intelligence and machine learning) in microbial enzyme production is also presented.

## 1. Introduction

As primary industries, both fisheries and aquaculture constitute essential sectors of the economy in many parts of the world [1]. The latest report from the Food and Agricultural Organization (FAO) stated that global fisheries and aquaculture production in 2022 reached an all-time record of 185.4 million tons of aquatic animals (i.e., fish, crustaceans and mollusks) [2]. Statistical data published in the same report also indicated that the aquaculture production in 2022 represented 51% of the total production, surpassing for the first time that from capture fisheries [2]. The recovery of capture fisheries, along with the expansion of aquaculture (which is currently the fastest-growing animal food production sector [3]), are believed to have positive impacts on aquatic animal production, with the latest projections predicting a 10% increase in production by 2032 [2].

The blue bio-economy aims to promote a sustainable utilization of fisheries resources for economic growth and development, along with the preservation of the ocean’s health [4]. However, achieving all the goals of the blue bio-economy could be challenging, mostly due to climate change and unsustainable practices in the fisheries and aquaculture sectors. For instance, climate change, on the one hand, has severe negative impacts on the ocean (such as ocean acidification as well as sea level and temperature rises) [5] and could constitute a hindering factor for sustainable ocean development [6]. The fisheries and aquaculture industries, on the other hand, still have not addressed the pressing need to operate in a sustainable manner. In this regard, it is known that fisheries and aquaculture production is not entirely used for human consumption. In fact, processing fish, shellfish and other marine animals results in significant amounts of waste, which, depending on the species, can reach as high as 50 to 70% of the total production [7,8]. Fishery and aquaculture waste is typically discarded at sea or in landfills and, in the best-case scenario, rendered into fishmeal and fish oil [9,10]. However, under the blue bio-economy concept, in which the sustainable utilization of the ocean and its resources is a priority, it is critical to minimize and/or eliminate waste generation [11]. Due to the abundance of value-added biomolecules (i.e., enzymes, proteins/peptides, polyunsaturated fatty acids, carotenoids, minerals and hydroxyapatite, as well as polysaccharides, including chitin and glycosaminoglycans [7,12,13]), fishery and aquaculture waste could be considered as a renewable source of highly valuable and industrially relevant bioproducts. The development of low-cost, green and circular processes for valorizing and/or upcycling this waste could decrease the pressure on marine ecosystems, avoid pollution and create sustainable wealth through revenue generation and job creation [7,11].

The aim of this review is to provide an overview of the major enzymes from fishery and aquaculture waste. This review also discusses the use of fishery and aquaculture waste as a substrate for the production of bacterial and fungal enzymes. In addition, the application of emerging technologies (i.e., artificial intelligence and machine learning) with respect to enzyme production using fishery and aquaculture waste as a substrate is presented.

## 2. Fishery and Aquaculture Waste

During the capture, farming and processing of fish, shellfish and other marine animals, substantial quantities of waste are produced. In capture fisheries, the waste mainly consists of by-catch (i.e., unwanted species that have lower commercial value than the target catch [9,14]). By-catch remains underutilized, largely due to several negatively viewed attributes in terms of color, flavor, texture, size and fat content [15]. Some examples of by-catch in commercial fish capture include butterfish (*Peprilus triacanthus*), which are a non-targeted species of the squid fishery [16], and Chinook salmon (*Oncorhynchus tshawytscha*), which are caught in the Bering Sea pollock fishery [17]. In shellfish fisheries (i.e., tropical shrimp), species that are highly susceptible to capture by trawls include ariid catfish (*Ariidae*), conger eels (*Congridae*), wrasse (*Labridae*) and eeltail catfish (*Plotosidae*) [18]. In the aquaculture industry, mortalities and discarded species are the major sources of waste [19].

**Figure 1:**
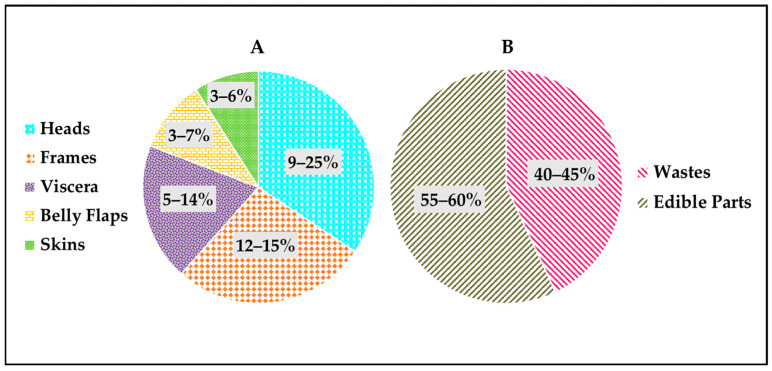
Approximate percentages of waste generated during processing of fish and shellfish: (**A**) waste generated during processing of fish, and (**B**) waste generated during processing of shellfish (i.e., shrimps and crabs).

When processing fish, shellfish and other marine animals into marketable seafood products, any parts that are not typically regarded as edible (i.e., not used for human consumption) are considered to be waste; this includes the heads, frames, tails, viscera, belly flaps, shells, etc. [20,21]. Figure 1 presents the approximate percentages of waste generated during the processing of fish and shellfish. In general, the percentages of waste relative to the whole fish (based on wet weight) are in the range of 9–25% for the heads, 3–6% for the skins, 12–15% for the frames, 5–14% for the viscera and 3–7% for the belly flaps [8,22,23,24]. For shellfish such as shrimps, the proportion of waste (heads, shells and tails) varies from 40 to 46% with respect to the whole shellfish’s wet weight [25]. For shrimp waste, the heads represent 72.5%, while the shells with tails represent 27.5% of the total waste [25]. For other shellfish species, such as crabs, the shells correspond to the waste generated during processing and represent about 55% of the whole crab’s wet weight [26].

Fishery and aquaculture waste (both by-catch and processing discards) are highly unstable due to their high organic matter and lipid content as well as their strong proteolytic enzyme activity. Recently, there has been a growing interest in valorizing fishery and aquaculture waste by recovering value-added products instead of converting it into low-value commodities (such as fishmeal, fish oil and compost/fertilizer) [27]. Numerous research studies indicated that fishery and aquaculture waste contains functional and bioactive compounds, such as a range of enzymes, proteins, lipids rich in omega-3 fatty acids, pigments, chitin, glycosaminoglycans, vitamins and other biomolecules with high market values [7,21,22]. Enzymes from fish, shellfish and other marine animals, in particular, have been reported to possess high catalytic activities at relatively low concentrations [7,22]. They also exhibit cold-adapted properties (i.e., active at low temperatures and unstable at higher temperatures) and remain stable over a wide range of pHs [7,22]. These properties make marine-derived enzymes very interesting from an economic point of view by lowering the energy requirement for enzyme-based processes [7].

## 3. Overview of Major Industrially Relevant Enzymes from Fishery and Aquaculture Waste

The internal organs of the fish, shellfish and other marine animals contain different types of enzymes. These marine-derived enzymes have been found to possess cold-adapted properties (i.e., active between 0 and 30 °C but unstable at temperatures higher than 50 °C [7,28]). The mechanisms of cold adaptation in marine enzymes have been recently reviewed by Khiari [7]. Briefly, cold adaptation is believed to be associated with a higher molecular flexibility that is not typically observed with mesophilic and thermophilic enzymes [7]. In addition, cold-adapted enzymes have been reported to have a smaller number of hydrogen bonds, a structure that is less densely packed, a higher surface hydrophilicity and a greater number of methionine residues [7]. It is worth noting that cold-adapted enzymes in fish species from the Northern Seas (i.e., Arctic region) are different than those from the Southern Seas (i.e., Antarctic region). In this regard, enzymes from Antarctic fish have been shown to be active at extremely low temperatures and are, in general, more stable in the presence of high salt concentrations and metal ions compared to those from Arctic fish [29,30].

Cold-adapted enzymes are generally preferred over mesophilic and thermophilic counterparts. Unlike mesophilic enzymes, cold-adapted enzymes are highly active at low temperatures (20–25 °C) and are able to preserve half of their maximum activity at even lower temperatures (0–10 °C); however, they are easily inactivated through moderate thermal treatment [30]. A number of studies investigated the kinetic properties of cold-adapted fish enzymes compared to mesophilic mammalian enzymes. Some examples of the kinetic properties of selected fish and mammalian enzymes are presented in Table 1. These kinetic studies proved that fish enzymes possess significantly higher catalytic efficiency at low temperatures compared to mammalian enzymes, as indicated by the k_cat_/K_m_ values.

Owing to their unique characteristics, cold-adapted enzymes from fish, shellfish and other marine animals have promising prospects for several industrial applications. This includes the food industry (i.e., processing at low temperatures slows the growth of pathogenic microorganisms [which subsequently increases food safety] and reduces the occurrence of oxidation reactions [which subsequently improves food quality]), detergent industry (i.e., cold-adapted enzymes are suitable for laundering in cold water), bioremediation (i.e., cold-adapted enzymes could be applied to decontaminate soils and waters in cold regions) as well as green chemistry (i.e., cold-adapted enzymes could be used for the synthesis of heat-labile chemicals) [34].

### 3.1. Proteases

Proteases (i.e., peptide-bond hydrolases) are enzymes that catalyze the cleavage of peptide bonds in both proteins and peptides. Peptidases are types of proteases that act on peptides and break them down into amino acids [35]. Generally, peptidases can either act on peptide bonds away from the protein termini (i.e., within the protein molecule, and in this case, the protease is termed endopeptidases) or cleave peptide bonds only near the C- or N-terminus (i.e., at a free terminus and, in this case, the protease is termed exopeptidases) [35]. Depending on the amino acid residue involved in the nucleophilic attack of the peptide-bond in the substrate, proteases are classified into six catalytic types: serine proteases, cysteine proteases, glutamic proteases, aspartic proteases, threonine proteases and metalloproteases [35,36].

In fish, proteolytic enzymes are mostly found in viscera, specifically in the stomach, pancreas, pyloric caeca and intestine [37], while in shellfish and other marine animals, digestive proteases are mainly found in the hepatopancreas [38]. Four major groups of proteolytic enzymes are found in fishery and aquaculture waste: aspartyl proteases, serine proteases, cysteine proteases and metalloproteases [39]. Aspartyl proteases (or acidic proteases) are found in the stomach and include pepsin, chymosin and gastricsin [40]. Serine proteases (or alkaline proteases) are found in the pyloric ceca and small intestine and include trypsin, chymotrypsin and elastase [7,40]. Cysteine proteases (or thiol proteases) are typically found in the intestine and include cathepsin [40]. Metalloproteases are proteases that require the presence of bound divalent cations to perform proteolytic hydrolysis and are usually found in muscle tissues [40] but have also been detected in the digestive tracts of marine animals (such as sea cucumber) [41], in fish viscera [42] as well as in fish heads [43].

**Figure 2:**
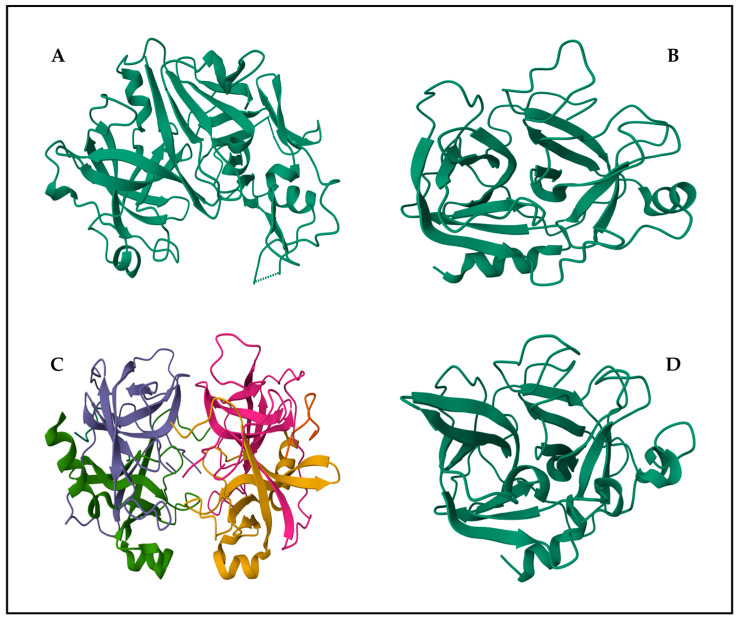
Structures of representative proteases: (**A**) pepsin, (**B**) trypsin, (**C**) α-chymotrypsin and (**D**) pancreatic elastase. The enzyme structures were obtained from the Research Collaboratory for Structural Bioinformatics Protein Data Bank—RCSB PDB (RCSB.org)—using PDB ID 1AM5 for pepsin from Atlantic cod [44], PDB ID 1HJ8 for trypsin from Atlantic salmon [45], PDB ID 4CHA for bovine α-chymotrypsin [46] and PDB ID 1ELT for elastase from North Atlantic salmon [47]. The different colors in subfigure (**C**) represent the different chains in α-chymotrypsin.

Figure 2 shows the structures of four representative proteases (pepsin, trypsin, chymotrypsin and elastase).

It is worth noting that the serine endopeptidases found in shellfish (i.e., crustacea) are called brachyurins [48]. Brachyurins comprise three distinctive types: (i) brachyurins Ia, which possess a wide specificity similar to that of trypsin, chymotrypsin, elastase and collagenase; (ii) brachyurins Ib, which possess a wide specificity but a reduced activity toward trypsin substrates; and (iii) brachyurins II, which are trypsin-like proteases [49].

In fishery and aquaculture waste, fish viscera [50], shellfish hepatopancreas [51] and the digestive tract in marine animals [41] represent the major sources of enzymes. Table 2 shows some examples of the proteases recovered from fishery and aquaculture waste.

### 3.2. Lipases

Lipases are lipolytic enzymes and comprise both triglyceride lipases and phospholipases [91]. Triglyceride lipases catalyze the hydrolysis of triglycerides, while phospholipases catalyze the hydrolysis of phospholipids [91]. In addition to lipid hydrolysis, lipases can also catalyze a number of other reactions, including esterification, transesterification, interesterification, acidolysis, aminolysis and alcoholysis [92,93]. Lipases are widely used in biotechnology due to their high specificity and milder reaction requirements [93].

During digestive lipolysis, two neutral lipid-hydrolyzing enzymes are released from the pancreas: pancreatic lipase and bile salt-dependent lipase [94]. Pancreatic lipases, also called triacylglycerol acylhydrolases, catalyze the hydrolysis of the carboxyl ester bonds of acylglycerols, converting them into diglycerides and subsequently into monoglycerides and free fatty acids [95]. Bile salt-dependent lipases, also called carboxyl ester lipase or cholesterol esterase, catalyze the hydrolysis of the carboxyl ester bonds of cholesterol esters and fat-soluble vitamin esters in addition to those of acylglycerols [94].

A number of studies have focused on lipases from fish, shellfish and other marine animals; however, marine lipases are relatively less studied compared to marine proteases. Table 3 shows some examples of the fishery and aquaculture waste used as sources of lipases.

Most recently, Liu et al. purified novel salt-tolerant, organic solvent-stable, and bile salt-activated lipase from the viscera of golden pompano (*Trachinotus ovatus*) [104]. Lipases have also been recovered from shellfish such as crab (*Carcinus mediterraneus*) [105,106], shrimp (*Penaeus vannamei*) [107,108] and the marine gastropod mollusk *(Hexaplex trunculus*) [109].

Among the phospholipases, phospholipase A_2_ (also called phosphatide 2-acyl-hydrolase), which catalyzes the hydrolysis of the ester bond at the *sn*-2 position of glycerophospholipids [110], is the most widely studied phospholipase [111]. The majority of phospholipase activity in fish has been observed in muscle, such as in pollock [112], Atlantic cod [113] and cod [111]. However, phospholipase activity has also been reported in fishery and aquaculture waste. For instance, phospholipase has been purified from the pyloric caeca of starfish (*Asterina pectinifera*) [114] and red sea bream (*Pagrus major*) [115]. Phospholipase has also been found in red sea bream’s gills [116] and hepatopancreas [117], as well as crab (*Carcinus mediterraneus*) digestive glands (i.e., hepatopancreas) [118].

### 3.3. Carbohydrases

A number of carbohydrases (i.e., carbohydrate-digesting enzymes) have been reported to be present in fish, and it is believed that the pyloric caeca, pancreas and intestinal mucosa are the main sources of these enzymes [119]. Specifically, the carbohydrate-digesting enzymes α-amylase, α-glucosidase, β-glucosidase and β-galactosidase have been detected in several fish species [120,121,122]. Amylase, the enzyme responsible for hydrolyzing starch to maltose, is by far the most studied amylolytic enzyme in fish. In this regard, Munilla-Mordn and Saborido-Rey assessed the amylase activity in the gut of sea bream (*Sparus aurata*), turbot (*Scophthalmus maximus*) and redfish (*Sebastes mentella*) [123]. Their findings indicated that the maximum activity of amylase from sea bream and turbot was around the neutral pH (7.0–7.5), while that from redfish was at acidic pH values (pH 4.5–5.0) [123]. The optimum temperature for the activity of the extracted amylases ranged between 35 and 45 °C for all the studied fish species [123]. Another study by Fish found that amylase was present in both perch and tilapia [124]. In tilapia, amylase was detected throughout the alimentary canal and mostly in the intestine, while in perch, amylase occurred in the pancreatic secretions [124].

Hidalgo et al. studied the amylase activity in six species of fish: rainbow trout (*Oncorhynchus mykiss*), sea bream (*Sparus aurata*), European eel (*Anguilla anguilla*), common carp (*Cyprinus carpio*), goldfish (*Carassius auratus*) and tench (*Tinca tinca*) [125]. Their results indicated that the omnivorous species presented higher amylase activity than the carnivores, and trout possessed the lowest amylase activity [125]. A similar observation of poor amylase activity in trout has been reported by Spannhof and Plantikow [126]. Skea et al. examined the amylolytic activity in three marine fish: butterfish (*Odax pullus*), silver drummer (*Kyphosus sydneyanus*) and marblefish (*Aplodactylus arctidens*) [127]. Their results also showed that the amylase activity was highest in the anterior gut wall tissue extracts from all three species of fish and decreased in the following order: marblefish followed by silver drummer and butterfish, respectively [127].

Amylase has also been reported in crustaceans. In this respect, amylase has been isolated from blue crab (*Portunus segnis*) viscera [128] and the midgut gland of three shrimp species (wild *Farfantepenaeus subtilis*, wild *Litopenaeus schmitti*, as well as farmed *Litopenaeus vannamei*) [129]. The results from the latter study indicated that the total amylolytic activity in farmed shrimp was three times higher than that from wild shrimp [129].

### 3.4. Chitinolytic Enzymes

Chitin is an abundant mucopolysaccharide in crustaceans [10]. Structurally, chitin is a β-(1,4)-linked polymer of N-acetyl-D-glucosamine (GlcNAc) [130]. Chitin-degrading enzymes hydrolyze chitin into GlcNAc through the synergistic action of endo-type chitinase (EC 3.2.1.14) and exo-type β-N-acetyl-hexosaminidase (EC 3.2.1.52) [131]. For instance, chitinases hydrolyze the β-1,4 glycosidic bonds of chitin to produce N-acetyl-chito-oligosaccharides (NACOs), which are subsequently degraded into GlcNAc units by β-N-acetyl-hexosaminidase [132]. High levels of chitinase activity were reported in fish stomachs [133,134], while low or negligible activities were observed in the pyloric caeca, intestines and livers of fish [132], whereas low levels of β-N-acetyl-hexosaminidase activity were reported in fish stomachs, but high levels were observed in the intestines and/or the pyloric caeca [132]. This suggests that chitinases start the hydrolysis of chitin in the stomach, which is further completely decomposed to soluble monomers of N-acetyl-D-glucosamine in the intestine and/or pyloric caeca by the action of β-N-acetyl-hexosaminidases [132]. The presence of both chitinase and β-N-acetyl-hexosaminidase in fish, shellfish and other marine animals could also suggest that these species are able to degrade chitin to penetrate prey exoskeletons as well as to turn them into nutrients (i.e., monosaccharide, N-acetyl-D-glucosamine) [134]. Chitinolytic activities have been observed in different parts of fish, shellfish and marine animals (stomach, intestine, pyloric caeca, liver, spleen, kidney, heart and gill [133]), which are generally considered to be waste [7]. Table 4 provides a summary of marine species that contain chitinolytic enzymes.

Chitosan, a β-1,4-linked polysaccharide consisting of glucosamine residues, is obtained through the enzymatic or chemical deacetylation of chitin [135]. To enhance its functionalities, chitosan is typically depolymerized to generate chitosan oligomers [136]. Chitosan depolymerization can be achieved through chemical (using acid) or enzymatic (using chitosanase) processes, with the enzymatic depolymerization process being preferable to the chemical depolymerization process [136]. The enzymatic depolymerization of chitosan is based on the hydrolytic action of chitosanases, which cleave the β-(1,4)-linked glycosidic bonds in chitosan. Chitosanases (EC 3.2.1.132) are largely found in microorganisms; however, they have also been detected in fish, shellfish and other marine animals. For example, Baehaki et al. [137], Affes et al. [138] and Yao et al. [139] reported the presence of chitosanases in the digestive tract of snakehead fish (*Channa striata*), the viscera of blue crab (*Portunus segnis*) and the digestive tract of sea cucumber (*Stichopus japonicus*), respectively.

**Table 4:** Examples of marine species containing chitinolytic enzymes.

Chitinolytic Enzyme	EC Number	Marine Species
Chitinase	EC 3.2.1.14	Silver croaker (*Pennahia argentatus*) [140,141], chub mackerel (*Scomber japonicus*) [140,142], Kuruma prawn (*Penaeus japonicus*) [143], Atlantic salmon (*Salmo salar*) [144], threeline grunt (*Parapristipoma trilineatum*) [145], fat greenling (*Hexagrammos otakii*) [142], marbled rockfish (*Sebastiscus marmoratus*) [146,147], Japanese sardine (*Sardinops melanostichtus*) [148], red sea bream (*Pagrus major*) [149], Japanese eel (*Anguilla japonica*) [150], Chinese black sleeper (*Bostrychus sinensis*) [151], red scorpionfish (*Scorpaena scrofa*) [152], Nile tilapia (*Oreochromis niloticus*) [153], coelacanth (*Latimeria chalumnae*) [154], red king crab (*Paralithodes camtschaticus*) [155], fiddler crab (*Uca pugilator*) [156], golden cuttlefish (*Sepia esculenta*) [157], squid (*Ommas trephes sloani pacificus*) [158], octopus (*Polypus dofleini*) [158], mussel (*Mytilus edulis*) [158] and octopus (*Eledone cirrhosa*) [158]
β-N-acetyl-hexosaminidase	EC 3.2.1.52	Marbled rockfish (*Sebastiscus marmoratus*) [147], cuttlefish (*Sepia esculenta*) [157,158], mussel (*Mytilus edulis*) [158], Japanese common squid (*Todarodes pacificus*) [158], flying squid (*Ommastrephes batrami*) [158], arrow squid (*Loligo bleekeri*) [158], brown sole (*Limanda herzensteini*) [132], European carp (*Cyprinus carpio*) [132], Pacific saury (*Cololabis saira*) [132], Atlantic bluefin tuna (*Thunnus thynnus*) [132], Pacific cod (*Gadus macrocephalus*) [132], Pacific herring (*Clupea pallasii*) [132], threeline grunt (*Parapristipoma trilineatum*) [132], chub mackerel (*Scomber japonicus*) [132], sardine (*Sardinops melanostictus*) [132], fat greenling (*Hexagrammos otakii*) [132], Japanese amberjack (*Seriola quinqueradiata*) [132], sea bass (*Lateolabrax japonicus*) [132] and red sea bream (*Pagrus major*) [132]

EC number: enzyme commission number.

### 3.5. Transglutaminases

Transglutaminases, also called protein-glutamine gamma-glutamyl-transferase, are Ca^2+^-dependent enzymes that belong to a class of transferases and catalyze the acyl-transfer between the glutamine residues and a wide variety of primary amines (i.e., catalyze the formation of cross-links between proteins) [159].

**Figure 3:**
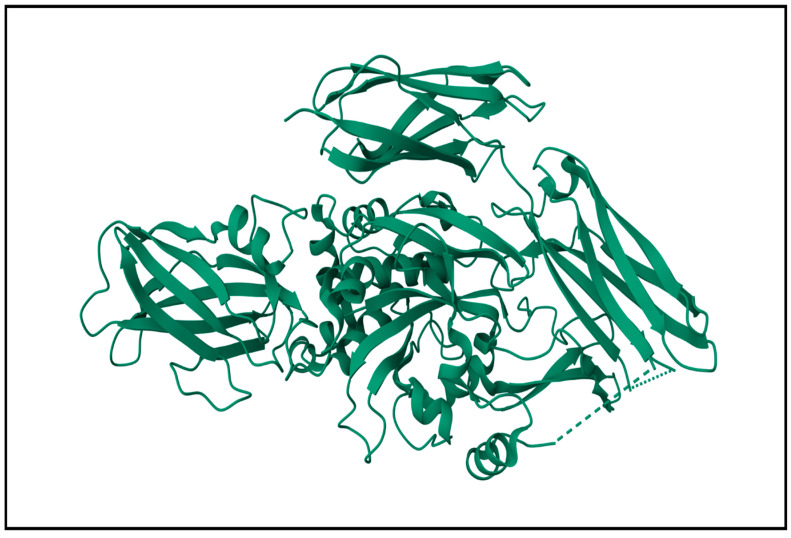
Structure of a representative transglutaminase. The enzyme structure was obtained from the Research Collaboratory for Structural Bioinformatics Protein Data Bank—RCSB PDB (RCSB.org)—using PDB ID 1G0D for transglutaminase from red sea bream [165].

Transglutaminase activity has been mostly found in the muscles of marine and freshwater fish as well as in the muscle tissues of shellfish and other marine animals [160,161]. In this regard, Nozawa et al. extracted and characterized transglutaminases from the muscles of six marine species: scallop (*Patinopecten yessoensis*), Botan shrimp (*Pandalus nipponensis*), squid (*Todarodes pacificus*), carp (*Cyprinus carpio*), rainbow trout (*Oncorhynchus mykiss*) and Atka mackerel (*Pleurogrammus azonus*) [160]. Binsi and Shamasundar isolated and characterized transglutaminases from four different fish species (bigeye snapper, Indian oil sardine, tilapia and common carp) [162]. Transglutaminases were also extracted from the tissues of Tropical tilapia (*Oreochromis niloticus*) [163] and Japanese oysters (*Crassostrea gigas*) [164]. Figure 3 shows the structure of a representative fish transglutaminase.

Presently, the majority of research on transglutaminases from marine species has focused on isolating the enzyme from muscle tissues. Only a limited number of studies investigated the recovery of transglutaminases from fishery and aquaculture waste. Examples of fishery and aquaculture waste from which transglutaminase was extracted include the red sea bream (*Pagrus major*) liver [166,167], walleye pollock (*Theragra chalcogramma*) liver [168], squid (*Todarodes pacificus*) gills [169] as well as horseshoe crab (*Limulus polyphemus*) hemolymph [170,171].

## 4. Utilization of Fishery and Aquaculture Waste for Production of Microbial Enzymes

The nitrogen source is by far the most expensive component of microbial growth media [172]. Fishery and aquaculture waste contains high protein levels and well-balanced amino acid profiles, making them ideal, low-cost sources of nutritional substrate for the growth of enzyme-producing microorganisms [173]. Numerous studies investigated the possibility of using fish processing waste as a substrate for bacterial and fungal enzyme production [174,175]. The available data indicate that fishery and aquaculture waste, either entirely (by-catch and mortality) or consisting of parts (heads, viscera, shells, etc.), has been successfully used as a substrate for enzyme production [174]. Many strains were reported to directly utilize fishery and aquaculture waste to meet their carbon and nitrogen requirements [176]. Examples of fishery and aquaculture waste that has been used as sources of nitrogen for bacterial and fungal enzyme production are listed in Table 5.

Microbial enzymes are typically produced through fermentation. This bioprocess can either be based on submerged fermentation (i.e., fermentation that occurs in a liquid medium with free water available for the microorganisms) or solid-state-fermentation (i.e., fermentation that occurs in a solid medium without or with very little free water available for the microorganisms) [209]. Several factors have been reported to affect the microbial enzyme production bioprocesses. This includes the microbial strain (which must be capable of producing a high yield of the target enzyme), the concentration and the type of nutrients in the culture medium, the pH, the temperature and other bioprocess parameters, such as agitation and aeration (especially for submerged-type fermentations) [209].

The global industrial enzyme market reached USD 6.1 billion in 2023 and is projected to attain USD 8.2 billion by 2030 [210]. Industries, including leather, paper, textile, biofuel, and, to a lesser degree, food industries, are among the major users of industrial enzymes [211]. Innovative research and development (R&D) efforts have been made, aiming at increasing enzyme production at a lesser cost by discovering new enzyme-producing microbial strains, improving the current available microbial strains and/or developing new microbial growth media [211]. Knowing that the culture media represent around 30% of the total cost of fermentation [212], the use of fishery and aquaculture waste as substitutes for the current commercial growth media could, therefore, significantly improve the economics of the entire enzyme production bioprocess. This approach not only upcycles fishery and aquaculture waste but also lowers enzyme production costs by offering low-cost microbial growth media (i.e., fishery and aquaculture waste-based media vs. industrially produced media). This, in turn, can boost the sustainability of these industries (i.e., seafood processing, enzyme production and enzyme biotechnology).

From the examples listed in Table 5, there is a clear indication that fishery and aquaculture waste is suitable as a substrate for microbial enzyme production. However, the utilization of microbial enzymes could face safety and regulatory concerns. For instance, as highlighted in Table 5, many enzymes are produced by microorganisms that could pose potential safety and health issues, such as being opportunistic pathogens to humans and/or non-human species, i.e., terrestrial and aquatic/marine animals. In addition, toxic substances (i.e., bacterial toxins and mycotoxins) could occur in the final enzyme preparations/isolates [213]. These drawbacks could make the approach of microbial enzyme production less attractive than the direct extraction of enzymes from the waste unless the enzyme-producing microorganisms are safe.

## 5. Application of Artificial Intelligence and Machine Learning in Microbial Enzyme Production Using Fishery and Aquaculture Waste as a Substrate

Artificial intelligence (AI) is a revolutionary technology in which computers have the ability to carry out intelligent tasks faster than humans can perform [214]. Machine learning (ML), a discipline of AI, relies on algorithms that receive and analyze data (input) and predict values (output) [215]. ML can be classified into four categories: supervised, unsupervised, semi-supervised and reinforcement learning. In supervised learning, the ML algorithms take direct feedback for the prediction [216]. In this case, the ML algorithm develops a function by mapping input variables from the training data into the desired output [217]. Artificial neural network (ANN), naive Bayes (NB) k-nearest neighbors (kNN), digital twin (DT), support vector machines (SVM), logistic regression (LR), etc., are among the most popular algorithms of supervised learning [216]. In unsupervised learning, ML algorithms do not take any feedback for the prediction [216], and in this case, ML algorithms discover patterns and structures in unlabeled data [218]. K-mean, as well as the self-organized model (SOM), principal component analysis (PCA), latent Dirichlet allocation (LDA), etc., are among the most well-known unsupervised learning algorithms [216]. The semi-supervised learning combines both labeled and unlabeled data to generate a function [219]. Reinforcement learning acts through a policy-based platform [217] and aims to achieve a goal by taking the appropriate measures to maximize the reward [220].

Traditionally, the optimization of microbial enzyme production was heavily based on the one-factor-at-a-time (OFAT) approach. However, OFAT can be inefficient and unreliable and can lead to false optimal conditions [221]. Recently, ML techniques have gained more relevance in the field of enzyme production and have been applied to determine the relationships between the input data (process conditions, such as the pH, temperature, substrate concentration, flow rates, etc.) and output variables (such as enzyme production yields) [215,222]. Artificial neural networks (ANN) and genetic algorithms (GAs) are among the most widely used AI-based techniques for the optimization of microbial enzyme production. ANNs are computer programs that mimic the learning ability of the human brain [223,224]. The ANN has two major advantages: the ability to work with large datasets and the ability to recognize complex patterns [225,226]. GAs, on the other hand, are random global search methods that imitate the process of mutation [226]. GAs simultaneously evaluate several parameters, and the search is based on the genetic selection principle (i.e., survival of the fittest) [227].

AI-based approaches have been applied to the production of bacterial and fungal enzymes using different agro-industrial wastes. For example, Fernández Núñez et al. investigated the production of amylases by *Rhizopus oligosporus* grown on wheat bran, sugarcane bagasse and soybean meal (either separately or combined) using an artificial intelligence approach [228]. Singh et al. optimized the production of glucansucrase by *Leuconostoc dextranicum* using an ANN and GA [229]. Bezerra et al. also optimized the production of cellulase by *Trichoderma stromaticum* grown on peach-palm wastes using an ANN and GA [230]. De Farias Silva et al. applied machine learning techniques (support vector machine (SVM) and ANN) to predict the yield of alginate lyase production by *Cunninghamella echinulate* grown on microalgae [231].

When it comes to the application of AI- and ML-based methods for the optimization of enzyme production using fishery and aquaculture waste as a substrate, only a limited number of studies have been carried out so far. For instance, Suryawanshi and Eswari optimized, through an ANN, the production of chitinase by *Thermomyces lanuginosus* using colloidal chitin extracted from shrimp shells [232,233]. Ekpenyong et al. optimized, through the application of the manta ray foraging optimization (MRFO) algorithm, the production of an acidic peptidase by an acidophilic *Bacillus cereus* strain grown using a mixture of yam peels and fish processing waste [234].

Despite the numerous advantages of AI- and ML-based techniques, the number of published studies related to applying these revolutionary approaches for improving enzyme production using fishery and aquaculture waste is still scarce. The difficulties of standardizing raw materials, as well as the lack of data due to the high diversity of marine species, could explain the scarcity of this type of study. In fact, fishery and aquaculture waste is commonly generated from multiple sources and has different properties, which creates challenges in producing standardized datasets for the ML models. Therefore, more studies are needed to fill this knowledge gap.

## 6. Concluding Remarks

There is an increasing need for the fisheries and aquaculture industries to adopt a zero-waste approach through the complete utilization of fisheries and aquaculture production, the reduction of waste generation and the valorization of the waste produced. Upcycling and/or valorizing fishery and aquaculture waste not only reduces its severe environmental impact but also increases its economic value. Within this context, the present review highlighted the major industrially relevant enzymes that are highly valuable bioproducts of fishery and aquaculture waste. The currently available scientific evidence indicates that fishery and aquaculture waste contains enzymes with unique characteristics compared to their mammalian counterparts. Specifically, marine-derived enzymes are known to possess greater enzymatic activity at lower temperatures combined with their reduced stability at higher temperatures. These properties are preferable from technological, economic and sustainability points of view since carrying out enzymatic processes at lower temperatures offers several advantages (i.e., cost reduction, energy savings, etc.). In addition, it has been confirmed that fishery and aquaculture waste represents low-cost, renewable sources of nutritional substrate for the growth of enzyme-producing microorganisms. However, enzymes produced by microorganisms could pose potential safety and health issues, making the microbial enzyme production approach unattractive unless safe enzyme-producing microorganisms are used. Extracting enzymes from fishery and aquaculture waste and/or turning the waste into a culture media for enzyme-producing microorganisms could potentially make these industries more circular, boost the bio-economy principles and further expand the field of microbial enzyme production. The application of artificial intelligence and machine learning technologies could also play a major role in advancing these fields. However, the real challenges facing the successful recovery/production of enzymes from fishery and aquaculture waste are associated with the inconsistent availability of the waste as well as the seasonal variations in enzyme activities.

## Figures and Tables

**Table 1:** Kinetic properties of cold-adapted fish enzymes compared to mesophilic mammalian enzymes at various temperatures *.

Enzyme	Source	Temperature (°C)	K_m_ (mM)	k_cat_ (s^−1^)	k_cat_/K_m_ (s^−1^ mM^−1^)	References
Trypsin	Bovine	4	0.30	0.21	0.70	[31]
	Porcine		0.96	1.04	1.08	
	Fish		0.037	0.90	24.3	
	Bovine	20	0.50	0.71	1.42	[31]
	Porcine		0.82	1.55	1.89	
	Fish		0.050	1.77	35.4	
	Bovine	37	0.72	1.35	1.84	[31]
	Porcine		0.91	2.72	2.99	
	Fish		0.068	2.69	39.6	
Elastase	Porcine	10	0.72	11.6	16.1	[32]
	Fish		0.79	24.6	31.1	
	Porcine	25	0.73	16.1	22.1	[32]
	Fish		1.02	44.1	43.2	

* Adapted from Smalås et al. [33]. K_m_: Michaelis constant; K_cat_: turnover number; k_cat_/K_m_: specificity constant.

**Table 2:** Proteases from fishery and aquaculture waste *.

Protease	Group	Marine Species
Pepsin	Aspartyl protease	Smooth hound (*Mustelus mustelus*) [52], European eel (*Anguilla anguilla*) [53], Atlantic cod (*Gadus morhua*) [54], sea bream (*Sparus latus*) [55], albacore tuna (*Thunnus alalunga*) [56,57], skipjack tuna (*Katsuwonus pelamis*) [57], tongol tuna (*Thunnus tonggol*) [57], mandarin fish (*Siniperca chuatsi*) [58] and pectoral rattail (*Coryphaenoides pectoralis*) [59]
Trypsin	Serine protease	Threadfin hakeling (*laemonema longipes*) [60], unicorn leatherjacket (*Aluterus monoceros*) [61], pectoral rattail (*Coryphaenoides pectoralis*) [62], zebra blenny (*Salaria basilisca*) [63], Japanese sea bass (*Lateolabrax japonicus*) [64], tongol tuna (*Thunnus tonggol*) [65], common kilka (*Clupeonella cultriventris caspia*) [66], skipjack tuna (*Katsuwonus pelamis*) [67], spotted mackerel (*Scomber australasicus*) [68], walleye pollock (*Theragra chalcogramma*) [69], Pacific saury (*Cololabis saira*) [70] and cuttlefish (*Sepia officinalis*) [71]
Chymotrypsin	Serine protease	Crucian carp (*Carassius auratus*) [72] and Pacific sardine (*Sardinops sagax caeruleus*) [73,74]
Elastase and collagenase	Serine protease	Haddock (*Melanogrammus aeglefinus*) [75], Atlantic herring (*Clupea harengus*) [75,76], flounder (*Paralichthys dentatus*) [75], sardine (*Sardina pilchardus*) [76], butterfly peacock bass (*Cichla ocellaris*) [77] and rough scad (*Trachurus lathami*) [77]
Brachyurin	Serine protease	Northern shrimp (*Pandalus borealis*) [78,79], Northern shrimp (*Pandalus eous*) [80], white shrimp (*Penaeus vannamei*) [81], greasyback shrimp (*Metapenaeus ensis*) [82], American lobster (*Homarus americanus*) [83], Caribbean spiny lobster (*Panulirus argus*) [49] and spiny lobster (*Panulirus interruptus*) [84]
Cathepsin	Cysteine protease	Atlantic cod (*Gadus morhua*) [85], sea cucumber (*Stichopus japonicus*) [86,87], rock bream (*Oplegnathus fasciatus*) [88], Atlantic herring (*Clupea harengus*) [89] and neon flying squid (*Ommastrephes bartramii*) [90]
Proteolytic enzymes	Metallo- protease	sea cucumber (*Stichopus japonicus*) [41], rainbow trout (*Oncorhynchus mykiss*) [42] and trout (*Salmo gairdnerii*) [43]

* This information (non-exhaustive) was compiled from a number of papers published during the past two decades (2004–2024).

**Table 3:** Examples of fishery and aquaculture waste used as sources of lipases.

Fishery and Aquaculture Waste	Marine Species	References
Pyloric caeca and pancreas	cod (*Gadus morhua*)	[96]
Pancreas	Atlantic salmon (*Salmo salar*)	[97]
	rainbow trout (*Oncorhynchus mykiss*)	[98]
Hepatopancreas	sea bream (*Pagrus major*)	[94]
	sardine (*Sardinella longiceps*)	[99]
Stomach, pyloric caeca, liver and intestine	rohu (*Labeo rohita*)	[95]
	Indian oil sardine (*Sardinella longiceps*)	[95]
	mullet (*Liza subviridis*)	[95]
	Indian mackerel (*Rastrelliger kanagurta*)	[95]
Stomach and intestine	tilapia (*Oreochromis niloticus*)	[100]
Viscera	catfish (*Clarias macrocephalus*)	[92]
	catfish (*Clarias gariepinus*)	[92]
	snakehead (*Channa stiata*)	[92]
	Nile tilapia (*Oreochromis niloticus*)	[92]
	Pacific sardine (*Sardinops sagax caerulea*)	[92,101]
	tuna species (*Euthynnusaffinis*)	[102]
Intestine, pancreas and pyloric caeca	milkfish (*Chanos chanos*)	[103]

**Table 5:** Examples of fishery and aquaculture waste used for production of microbial enzymes.

Fishery and Aquaculture Waste	Microorganisms	Enzymes	References
Fish heads and viscera	*Pseudomonas aeruginosa* *	Protease	[177]
	*Bacillus subtilis*	Protease	[178]
Fish viscera	*Vibrio anguillarum* *	Protease	[179]
	*Vibrio splendidus* *	Protease	[179]
Fish heads	*Streptomyces speibonae*	Protease	[180]
	*Paenibacillus elgii*	Protease	[181]
Shrimp shells	*Bacillus subtilis*	Protease	[182]
	*Chryseobacterium indologenes* *	Protease	[183]
	*Bacillus subtilis*	Chitosanase	[184]
	*Pseudomonas* *	Chitinase	[185]
	*Pseudomonas* *	Chitosanase	[185]
	*Bacillus cereus* *	Protease	[186]
	*Bacillus cereus* *	Chitinase	[186]
	*Serratia marcescens* *	Protease	[187]
	*Serratia marcescens* *	Chitosanase	[187,188]
	*Lecanicillium muscarium*	β-N-acetyl-hexosaminidase	[189]
	*Paenibacillus mucilaginosus*	Chitosanase	[190]
Shrimp and crab shells	*Monascus purpureus* *	Protease	[191]
	*Bacillus firmus*	Protease	[192]
	*Bacillus amyloliquefaciens*	Chitinase	[193]
	*Monascus purpureus*	Chitinase	[194]
	*Bacillus cereus* *	Chitinase	[195]
Cuttlefish by-products	*Bacillus licheniformis*	Protease	[176]
	*Bacillus subtilis*	Protease	[176]
	*Pseudomonas aeruginosa* *	Protease	[176]
	*Bacillus cereus* *	Protease	[176]
	*Vibrio parahaemolyticus* *	Protease	[176]
Squid pens	*Lactobacillus paracasei*	Protease	[196]
	*Serratia ureilytica* *	Protease	[197]
	*Lentzea*	Chitosanase	[198]
	*Serratia ureilytica* *	Chitinase	[197]
	*Paenibacillus* sp.	Protease	[199]
	*Paenibacillus* sp.	Chitosanase	[199,200]
	*Paenibacillus elgii*	Chitosanase	[201]
Fish by-products	*Staphylococcus epidermidis* *	Lipase	[202]
	*Bacillus cereus* *	Protease	[203]
Fish and shellfish wastes	*Staphylococcus xylosus*	Lipase	[204]
Hydrolyzed fish wastes	*Rhizopus oryzae*	Lipase	[172]
	*Bacillus mojavensis*	Protease	[205]
Shrimp by-products	*Purpureocillium lilacinum* *	Chitosanase	[206]
Chitinous fishery wastes	*Paenibacillus* sp.	Chitinase	[207]
Shrimp waste silage	*Verticillium lecanii*	β-N-acetyl-hexosaminidase	[208]

* Indicates that the microorganism could pose potential health and safety issues.
